# N332-Directed Broadly Neutralizing Antibodies Use Diverse Modes of HIV-1 Recognition: Inferences from Heavy-Light Chain Complementation of Function

**DOI:** 10.1371/journal.pone.0055701

**Published:** 2013-02-19

**Authors:** Marie Pancera, Yongping Yang, Mark K. Louder, Jason Gorman, Gabriel Lu, Jason S. McLellan, Jonathan Stuckey, Jiang Zhu, Dennis R. Burton, Wayne C. Koff, John R. Mascola, Peter D. Kwong

**Affiliations:** 1 Vaccine Research Center, National Institute of Allergy and Infectious Diseases, National Institutes of Health, Bethesda, Maryland, United States of America; 2 Department of Immunology and Microbial Science and IAVI Neutralizing Antibody Center, The Scripps Research Institute, La Jolla, California, United States of America; 3 Ragon Institute of MGH, MIT, and Harvard, Cambridge, Massachusetts, United States of America; 4 International AIDS Vaccine Initiative (IAVI), New York, New York, United States of America; University of Massachusetts Medical Center, United States of America

## Abstract

Dozens of broadly neutralizing HIV-1 antibodies have been isolated in the last few years from the sera of HIV-1-infected individuals. Only a limited number of regions on the HIV-1 spike, however, are recognized by these antibodies. One of these regions (N332) is characterized by an *N*-linked glycan at residue 332 on HIV-1 gp120 and is recognized by antibody 2G12 and by the recently reported antibodies PGT121-137, the latter isolated from three donors. To investigate the diversity in mode of antibody recognition at the N332 site, we used functional complementation between antibody heavy and light chains as a means of assessing similarity in mode of recognition. We examined a matrix of 12 PGT-heavy chains with each of 12 PGT-light chains. Expression in 96-well format for the 144 antibodies (132 chimeric and 12 wild-type) was generally consistent (58±10 µg/ml). In contrast, recognition of HIV-1 gp120 was bimodal: when the source of heavy and light chains was from the same donor, recognition was good; when sources of heavy and light chains were from different donors, recognition was poor. Moreover, neutralization of HIV-1 strains SF162.LS and TRO.11 generally followed patterns of gp120 recognition. These results are consistent with published sequence, mutational, and structural findings, all of which indicate that N332-directed neutralizing antibodies from different donors utilize different modes of recognition, and provide support for a correlation between functional complementation of antibody heavy and light chains and similarity in antibody mode of recognition. Overall, our results add to the growing body of evidence that the human immune system is capable of recognizing the N332-region of HIV-1 gp120 in diverse ways.

## Introduction

The human immune system can generate antibodies that effectively recognize HIV-1- if given sufficient time: although broadly neutralizing antibodies are not elicited within the first year of HIV-1 infection, they do appear after two or more years in roughly 20% of HIV-1-infected donors [1]–[6]. Indeed, mining the B cell repertoire of HIV-1-infected donors for broadly neutralizing antibodies has proven extraordinarily fruitful (reviewed in [7]). Beginning with antibodies PG9 and PG16 from the IAVI Protocol G cohort [8], dozens of additional broadly neutralizing antibodies have been characterized [9]–[13] and sequences for hundreds more determined [11], [12] (reviewed in [14]). In contrast, prior to 2009, only four broadly neutralizing antibodies had been discovered: 2F5, 2G12, 4E10 and b12, all isolated in the mid 1990’s [15]–[20].

Although characterization of this plethora of new antibodies is continuing, initial observations suggest that the new antibodies target just a few regions on HIV-1 [21], and often in similar ways. For example, numerous broadly neutralizing antibodies have been identified that target the site of CD4-receptor binding on the HIV-1 gp120 envelope glycoprotein, with antibodies from multiple individuals converging on the same mode of “VRC01-like” recognition, which utilizes heavy chain mimicry by antibody for the recognition of the CD4 receptor [10]–[12]. Another site on HIV-1 gp120 targeted by broadly neutralizing antibodies is characterized by an N-linked glycan at residue 332. Antibodies against the N332 site include the prototype 2G12 antibody [17] as well as twelve recently identified antibodies named PGT121-137 from three elite neutralizers (IAVI protocol G donors, 17, 36 and 39) [13]. Neutralizing activity directed towards the N332 site is one of the first as well as one of the most commonly observed activities with breadth identified in longitudinal studies of HIV-1 infected individuals [22], and N332-directed antibodies with broad neutralization were shown to develop in a macaque after less than one year of simian/human immunodeficiency virus infection [23].

Do N332-directed antibodies from different donors use many modes of HIV-1 recognition, or do they, like the VRC01-like antibodies, converge on a common mode? By common mode of recognition, we mean that similar residues (e.g. at equivalent positions on complementarity-determining regions) on different antibodies would interact with the same residues on gp120. Crystal structures of N332-directed antibodies 2G12 [24] and PGT128 [25] identify at least two different modes of recognition, but the modes of recognition for antibodies PGT121-123 and PGT135-137, from Protocol G donors 17 and 39, respectively, were not defined. Subsequent to the submission of this manuscript, the structure for the PGT121 was described [26]; and we have included an analysis of this structure by homology modeling. Here we exploit the ability of the heavy and light chains from one antibody to complement the heavy and light chains of a different antibody as long as the two antibodies share the same mode of antigen recognition. We used 96-well microplate-formatted expression to efficiently produce a complete matrix of PGT121-137 heavy and light chain chimeras. We assessed antibody function by analyzing gp120 recognition and HIV-1 neutralization. The results provide insight into the diversity of antibody recognition at the N332 site of HIV-1 gp120.

## Materials and Methods

### Phylogenetic Analysis

The multiple sequence alignment and phylogenetic tools implemented in ClustalW [27] were used to calculate diversity for PGT121-137 and CD4-binding site-directed antibodies. Briefly, a multiple sequence alignment was first constructed for each set of antibody sequences and then subjected to the neighbor-joining (NJ) method in CLUSTALW2 using the “Phylogenetic trees” option [28]. The nucleotide distance was calculated as the percent divergence between all pairs of sequences from the multiple alignment, and the resultant tree displayed as a radial polygram.

### Construction of Plasmids Encoding Heavy and Light Chains for Antibodies PGT121-137

Sequences for each of the heavy and light chains from PGT121-137 [13] were codon optimized for expression in Homo sapiens, synthesized and cloned into the pJ603 mammalian expression vector (DNA 2.0, CA). The HRV 3C protease recognition sequence (LEVLFQGP) was inserted into the hinge region of heavy chains to allow for efficient production of the antigen-binding fragment. Plasmids containing either the heavy or the light chain were prepared by standard methods (DNA 2.0, CA, or GeneImmune, MD).

### Transient Transfection Expression of Antibodies in 96-well Microplates

96-well plate-formatted mammalian cell culture has been mainly used in cell-based assays [29], and high-throughput expression of mammalian proteins is generally accomplished through 96-well plate-formatted bacterial cell culture [30]. In order to achieve high-throughput expression of a high-titer human antibody matrix, we formulated our own 96-well microplate-formatted transient transgene expression approach as follows. HEK 293T cells (Invitrogen, CA) were thawed and incubated with growth medium (Dulbecco’s Modified Eagle Medium with 10% Fetal Bovine Serum and 1% streptomycin-penicillin) (Invitrogen, CA) at 37°C, 5% CO_2_, until the cells reached logarithmic physiological growth. 24 hours prior to DNA-transient transfection, 100 µl of physiologically growing cells was seeded in each well of a 96-well microplate at a density of 2.5×10^5^ cells/ml in Dulbecco’s Modified Eagle Medium supplemented with 10% Ultra-Low IgG Fetal Bovine Serum and 1×-Non-Essential Amino Acids (Invitrogen, CA), and incubated at 37°C, 5% CO_2_ for 20 h. Two hours prior to transfection, 100 µl of spent medium from each well was replaced with 65 µl of fresh medium.

**Figure 1:**
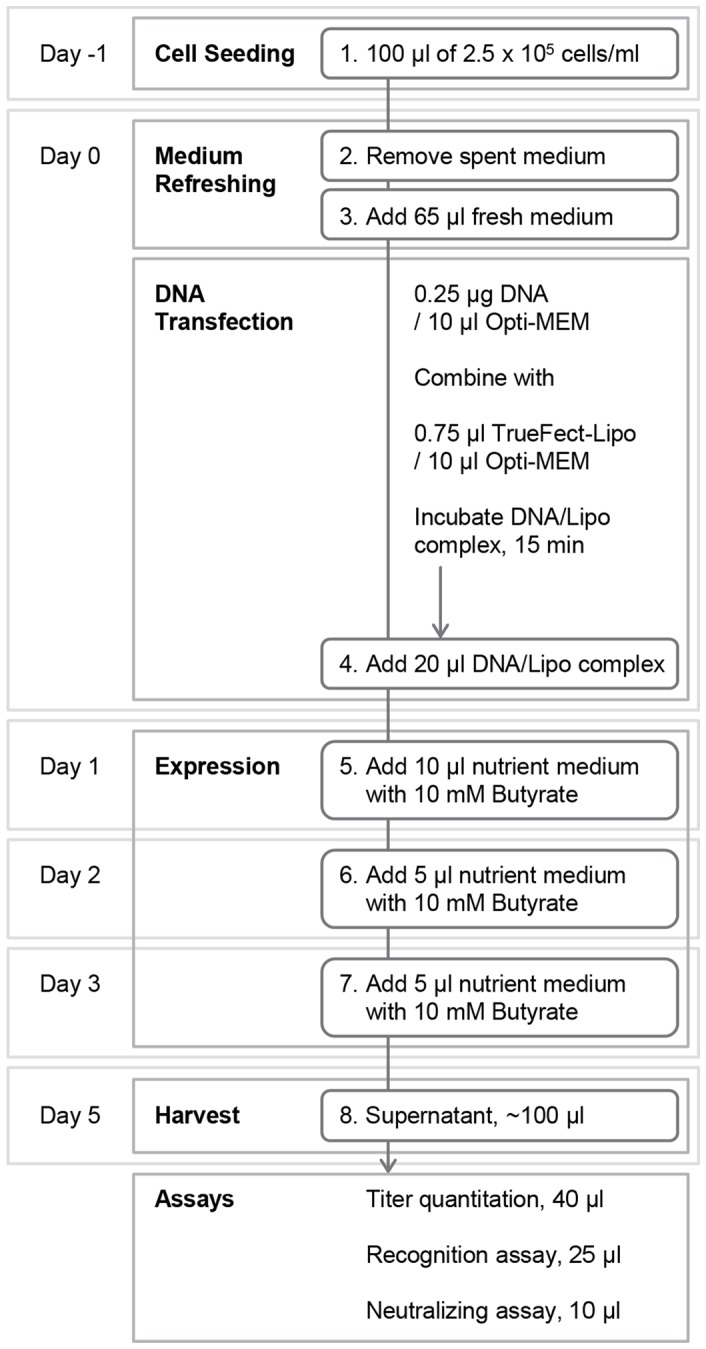
Schematic of 96-well microplate-formatted high-throughput antibody expression. Starting with cell seeding, the entire protocol takes 7 days. At the end, the supernatant in each well is directly used for 96-well microplate-formatted assays.

DNA-TrueFect-Max complexes were used for transfection, and these were prepared by mixing 0.25 µg DNA (0.125 µg of heavy chain plasmid combined with 0.125 µg of light chain plasmid) in 10 µl of Opti-MEM transfection medium (Invitrogen, CA) with 0.75 µl of TrueFect-Max (United BioSystems, MD) in 10 µl of Opti-MEM, and incubating for 15 min prior to transfection. 20 µl of the complex was added into each well and mixed with growing cells, and the 96-well plate was incubated at 37°C, 5% CO_2_. One day post-transfection, 10 µl of enriched medium (High Glucose Dulbecco’s Modified Eagle Medium plus 25% Ultra-Low IgG Fetal Bovine Serum, 2× Non-Essential Amino Acids, 2× glutamine and 10 mM butyrate) was added to each well, and on days two and three post-transfection, 5 µl of enriched medium was added into each well. On days three to five post-transfection, the culture was exposed to oxygen in the sterilized air once per day. After day five post-transfection, the IgG titer in the supernatant in each well of 96-well microplate was quantified using an Octet RED384 (ForteBio, CA). These details are summarized in Figure 1.

### Quantification of Antibody Expression

Anti-human IgG Fc biosensors (ForteBio, CA) were presoaked in 1× Kinetics buffer (ForteBio, CA). 40 µl of 96-well microplate supernatant was diluted in 160 µl of PBS for analysis, with 40 µl of non-transfection culture medium diluted in 160 µl of PBS as a negative control and with known concentrations of VRC01 IgG used as standards. IgG titers in the supernatants were quantified by Octet (ForteBio, CA), and standard curves generated using Octet data analysis software (ForteBio, CA). Unless explicitly stated otherwise, all samples were tested with anti-human IgG biosensors with an assay time of 200 s and a flow rate of 400 rpm. The IgG titers were automatically calculated using the Octet analysis software.

### Small-scale Protein Production

100 µg of light chain plasmid DNA and 100 µg of heavy chain plasmid DNA in 10 ml of Opti-MEM transfection medium (Invitrogen, CA) were mixed with 500 µl of TrueFect-Max (United BioSystems, MD) in 10 ml of Opti-MEM for 15 minutes before the DNA- TrueFect-Max complex was added into 225 ml of FreeStyle 293F cells (1.2×10^6^ cells/ml) in a 500-ml shaking flask. The transfected cells were returned to suspension incubation for 1 day, and then the culture was fed with 25 ml of CellBoost-5 (HyClone, Logan, UT). 6 days post transfection, supernatant was harvested by centrifugation and filtered through 0.22 µm filter. Antibody was purified through a column of Protein A Plus agarose (Thermo, IL), dialyzed against PBS and analyzed with SDS-PAGE.

### Assessment of HIV-1 gp120 Recognition by ELISA

200 ng of BaL, YU2 and CAAN gp120 monomeric proteins, expressed in 293T cells and purified over 17b-affinity column, were coated onto Maxisorb (Fisher Scientific, PA) 96-well ELISA plates in PBS and incubated overnight at 4°C. The wells were then washed once with PBS +0.2% Tween 20, and 300 µl of blocking buffer (PBS with 5% w/v dry milk) was added per well for 2 hours at room temperature (RT). 25–50 µl of antibody supernatant was added to the wells for 1 hour at RT and the wells were washed 5 times in PBS +0.05% Tween 20. Horseradish peroxidase (HRP)-conjugated goat anti-human IgG (H+L) antibody (Jackson ImmunoResearch Laboratories Inc., PA) at 1∶5,000 was added for 1 hour at RT to each well. The wells were then washed 5 times in PBS +0.05% Tween 20. The wells were developed using 3,3′,5,5′-tetramethylbenzidine (TMB) (Kirkegaard & Perry Laboratories, MD) at RT for 10 min and the reaction stopped with 180 mM HCl. The readout was measured at a wavelength of 450 nm. All samples were performed in duplicate.

### Neutralization of HIV-1

HIV-1 neutralization was assessed using single-round of replication Env-pseudoviruses and TZM-bl target cells as previously described [31]–[33]. 10 µl of undiluted supernatants containing IgG from transient transfection was added to 40 µl of pseudovirus, producing a 1∶5 final dilution of the supernatant. The percent of virus neutralization was calculated based on control well with no antibody.

### Structural Predictions of Chimeric Antibodies

Structural models of heavy and light chain of Mabs PGT121-137 were constructed using sequence alignment and Nest, a homology modeling program based on rigid-body optimization [34], as described previously [35]. The structures of PGT128 (PDB accession code 3TYG [25]) and 2G12 (PDB accession code 1OM3 [24]) were each used as templates for the structure prediction. We also included in our analysis the recently published PGT121 (PDB accession code 4FQ1 [26]). The model quality was evaluated by using the DFIRE scoring algorithm [36].

## Results

### Phylogenetic Analysis of PGT121-137 Antibodies

Functional complementation between different heavy chains or light chains depends on their structural and functional similarity. As phylogenetic analysis provides a rough estimate of similarity, we carried this out on the PGT121-137 antibodies. The heavy chains of antibodies PGT121-137 all derive from the IGHV4-family, with those from donors 36 and 39 derived from exactly the same V- and J-alleles (IGHV4-39*07 and IGHJ5*02), while the light chains of these antibodies in each of the three separate donors derives from a different genomic V-gene (IGLV3-21*02, IGLV2-8*01 and IGKV3-15*01) [13]. In addition, all 12 antibodies from each of the three donors were substantially affinity matured, with 15–23% of heavy chain nucleotides altered from germline.

**Figure 2:**
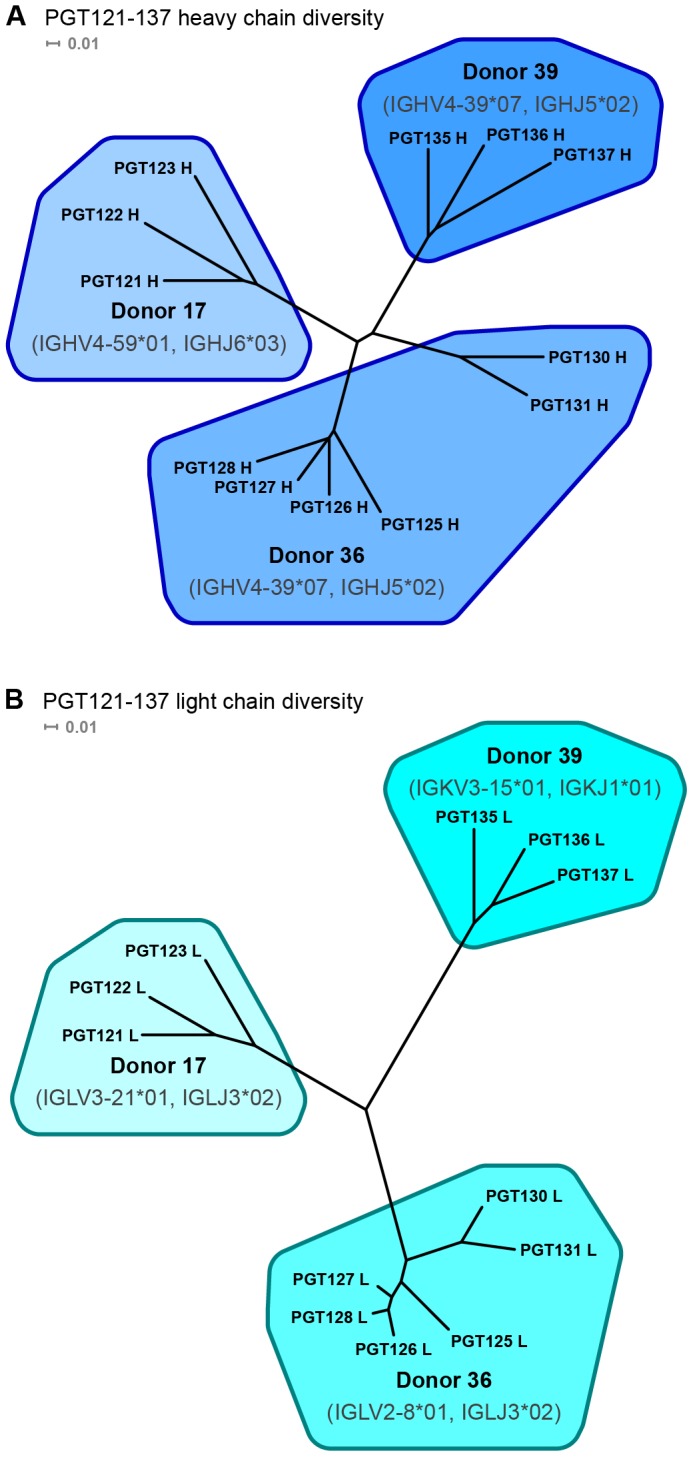
Heavy and light chain nucleotide sequence diversity derived from phylogenetic analysis of PGT121-137 antibodies. Neighbor-joining (NJ) method was used to calculate the dendrogram. Antibody branches are color-coded according to donors with putative V and J genes labeled under the donor specification.

With the heavy chains, phylogenetic analysis placed the 12 PGT121-137 antibodies into four heavy chain groups (Figure 2A), with each of the donor antibodies forming distinct phylogenetic groups, and antibodies PGT125-128 and PGT130-131 from donor 36 forming two separate groups. Also, despite having the same V/J origin, the heavy chains of antibodies from donors 36 and 39 evolved to be as distinct from each other as they are from antibodies PGT121-123 from donor 17.

With the light chain, phylogenetic analysis segregated the PGT121-137 antibodies into three light chain groups (Figure 2B). The light chains of PGT125-128 were slightly segregated from those of PGT130-131, but were much less divergent than their respective heavy chains.

### 96-well Expression of a Matrix of PGT121-137 Heavy and Light Chains

To express the full matrix of PGT121-137 heavy and light chains (comprising 12 wild-type and 132 heavy/light chimeras), we utilized 96-well microplate-formatted transient transgene expression. Plasmids encoding the PGT121-137 heavy and light chains were used to transiently transfect 293F cells in each well, and expression levels of IgG were quantified for the entire matrix by means of an anti-human antibody coupled to an Octet sensor (Figure S1). To facilitate the downstream neutralization assays, we enhanced antibody yield through optimization of cell growth, expression, and transient transfection parameters as demonstrated by the 14.8-fold increase in the yield of VRC01 antibody expression (Figure S2). Generally PGT antibody expression was in the 50–70 µg/ml range (58±10 µg/ml for the entire matrix, Figure S1). Chimeras containing the PGT130 light chain, however, displayed reduced expression (as low as 14 and 17 µg/ml for the heavy chains of PGT131 and PGT125 respectively). In contrast, chimeras containing the PGT135 heavy chain expressed at 80–90 µg/ml. The effectiveness of heavy and light chain co-transfection between inter-donor or intra-donor was evaluated by analysis of the antibody expression levels. The results from small scale expression of a select number of chimeric antibodies and SDS-PAGE analysis indicated that the expression of light chains or heavy chains in inter-donor chimeric Abs (PGT121-H/PGT128-L, PGT121-H/PGT135-L, PGT128-H/PGT121-L and PGT135-H/PGT121-L) were similar to those in intra-donor chimeric Abs (PGT121-H/PGT121-L, PGT121-H/PGT122-L, PGT122-H/PGT121-L) suggesting that co-transfection of a pair of light and heavy chains between inter-donor or intra-donor was equally effective (Figure S3) and that there was no major disruption at the interface between heavy and light chain.

### Recognition of HIV-1 gp120

**Figure 3:**
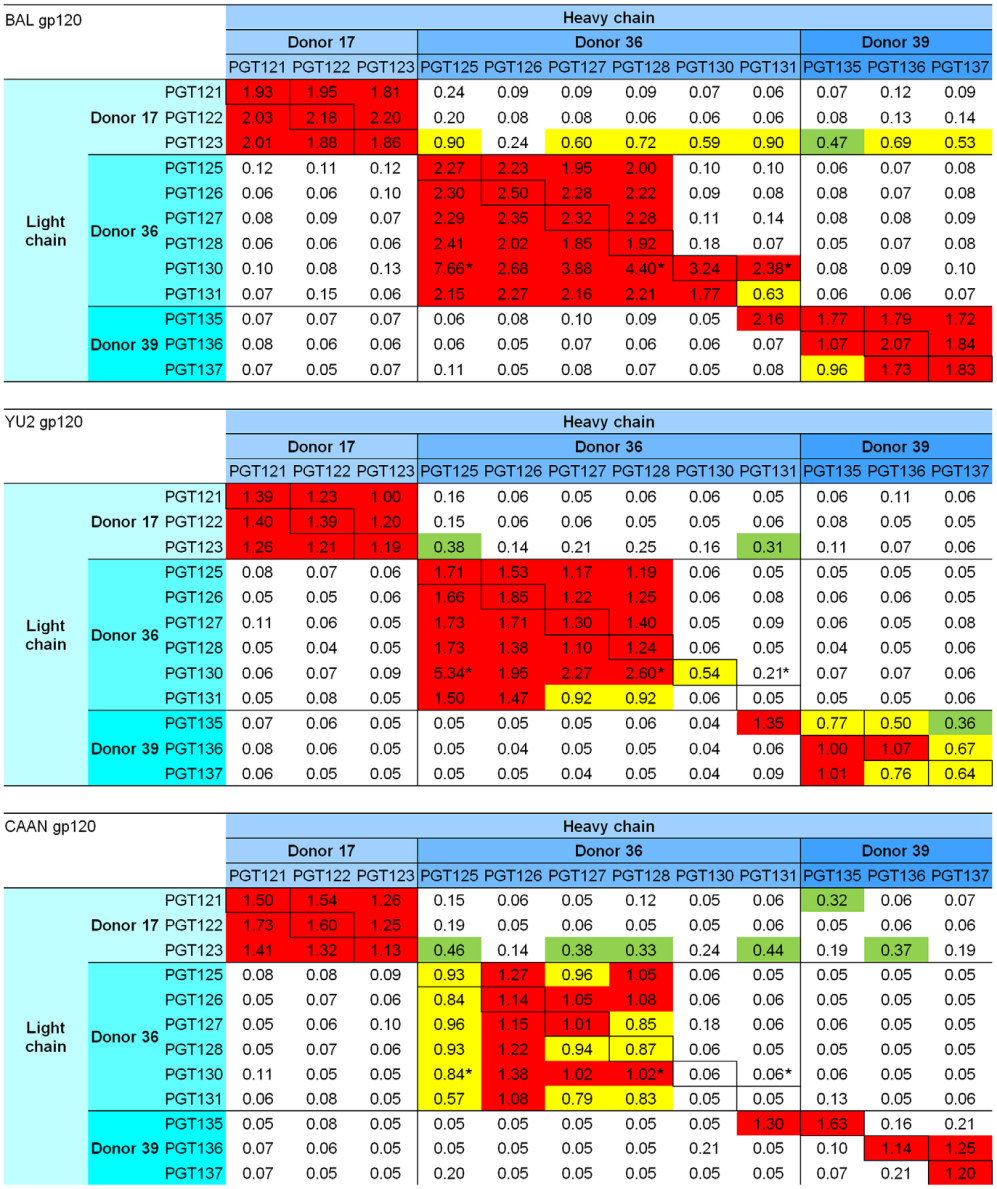
ELISA binding to HIV-1 gp120 by PGT121-137 antibodies and chimeric variants in units of OD_450_ **nm.** Strong binding is coded in red (>1.0 OD450 nm), intermediate in yellow (0.5–1.0 OD450 nm) and weak in green (0.3–0.5 OD450 nm). Each reported value is the average from three individual measurements, with the absorbance normalized by the expression titer. Note that in cases when the expression level of the antibodies was more than 2-fold different from the average, the normalization is probably not accurate and these numbers have been marked with a star. For comparison, the raw ELISA data has been added as Figure S4.

To assess the ability of the PGT antibodies (wild-type and chimeric) to recognize HIV-1 gp120, ELISAs were carried out with the supernatants of the transiently transfected cells using three full-length HIV-1 gp120s from the primary isolates BaL, YU2, and CAAN. In general, recognition was bimodal: with wild-type antibodies or with chimeras of heavy and light chains from the same donor (intra-donor complementation), recognition was high (1.93±1.21 OD_450_ for BaL, 1.10±0.86 OD_450_ for YU2 and 0.80±0.53 OD_450_ for CAAN); with chimeras of heavy and light chains from different donors (inter-donor complementation), recognition was low (0.16±0.28 OD_450_ for BaL, 0.09±0.15 OD_450_ for YU2 and 0.11±0.16 OD_450_ for CAAN) (Figure 3, Figure S4).

Three exceptions to this intra-donor/inter-donor trend were observed. First, recognition by antibodies with the heavy chain of PGT130 and PGT131 was generally weak (inter and intra-donor) (0.55±0.90 OD_450_ for BaL, 0.15±0.28 OD_450_ for YU2 and 0.14±0.26 OD_450_ for CAAN), even for the wild-type antibodies (0.30±0.35 OD_450_ for YU2 and 0.06±0.01 OD_450_ for CAAN). The reduced recognition of BaL gp120 by PGT131 was previously reported [13], as was the reduced recognition of CAAN gp120 by PGT130 and 131 [13], so the reduced PGT130/131 recognition likely reflects an inability of these antibodies to recognize the gp120s tested. Note that chimeric antibodies between PGT130 and PGT131 show good binding to BaL when produced from their own heavy and light chains. They also have good binding to BaL when PGT130 and PGT131 light chains are paired with PGT125-128 heavy chains from the same donor 36. The binding to BaL, however, is lost when PGT130-131 heavy chains are paired with PGT125-128 light chains. This might indicate that not all intra-donor chimeric antibodies complement. Second, recognition from inter-donor chimeras containing the light chain of PGT123 was intermediate (0.63±0.21 OD_450_ for BaL, 0.19±0.1 OD_450_ for YU2 and 0.30±0.12 OD_450_ for CAAN). Surprisingly, this medium level of recognition also occurred with the heavy chains of PGT130 and PGT131, with the recognition of CAAN by the PGT123 light chain/PGT130-131 heavy chain chimeras actually exceeding the recognition by wild-type PGT130/PGT131 (0.34±0.14 OD_450_ for the chimeras versus 0.055±0.01 OD_450_ for the wild-type). Third, recognition by the PGT131 heavy/PGT135 light chimera was strong (1.60±0.48 OD_450_). The strong recognition by this chimera represents the sole strong recognition by any of the 90 inter-donor chimeric antibodies, which showed an average recognition of 0.12±0.20 OD_450_ against the three gp120s.

### Neutralization of HIV-1 Isolates SF162 and TRO.11

**Figure 4:**
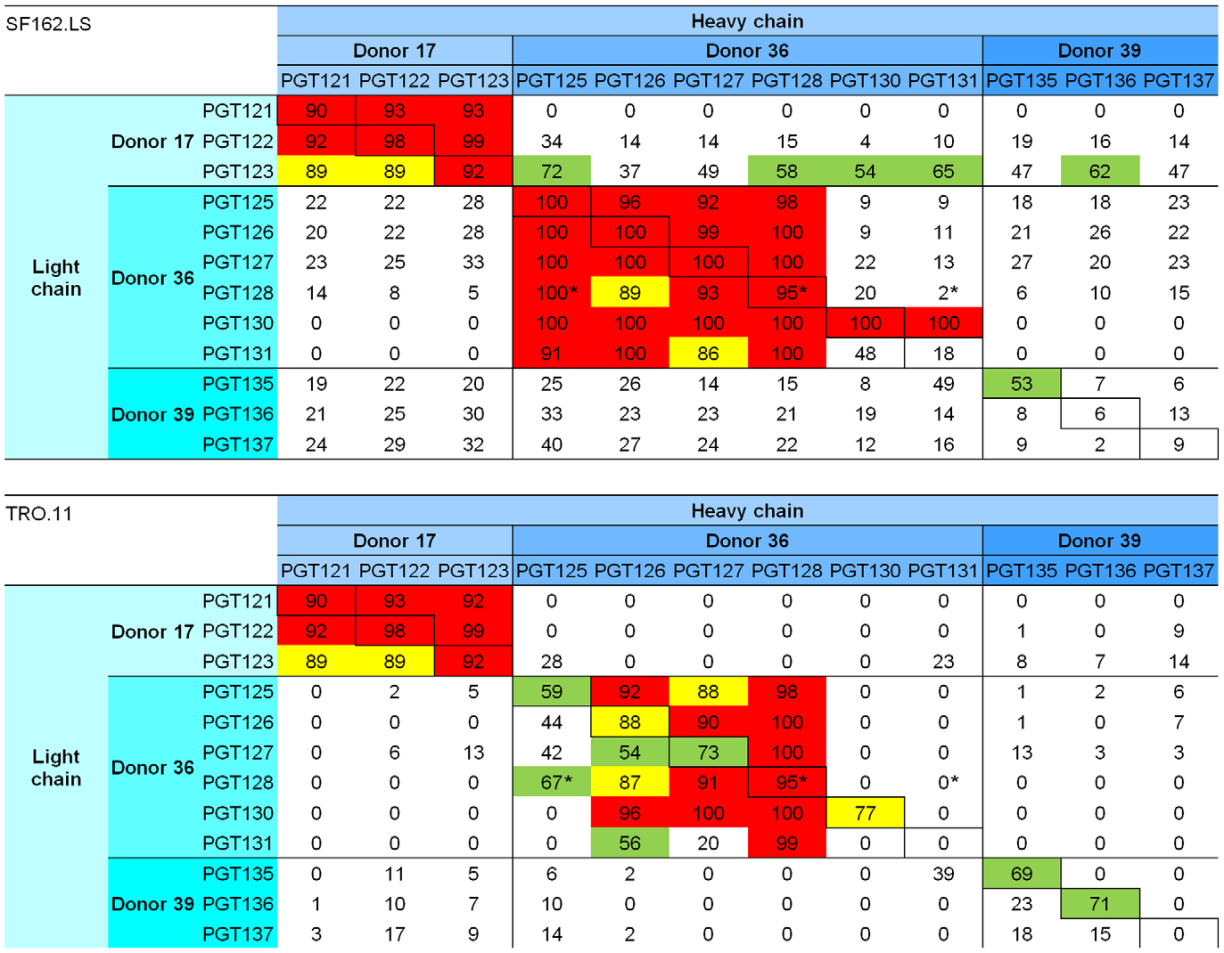
Neutralization by PGT121-137 antibodies and chimeric variants. Strong neutralization is coded in red (>90%), intermediate in yellow (75–90%) and weak in green (50–75%). Neutralization data were generated in single neutralization experiments, with each test sample analyzed in duplicate to determine the percent decrease in virus growth (neutralization) as compared and normalized to the control wells without antibody expression (defined as 0% neutralization). Each antibody was assayed at a 1∶5 final dilution with virus added. Neutralization results were normalized by the relative antibody expression, with the maximum neutralization defined to be 100%. Note that in cases when the expression level of the antibodies was more than 2-fold different from the average, the normalization is probably not accurate and these numbers have been marked with a star. For comparison, the raw neutralization data has been added as Figure S5. Supernatants expressing antibody 17b showed 0% neutralization with HIV-1 TRO.11 and 27% neutralization with HIV-1 SF162.LS; supernatants expressing VRC01 showed 100% neutralization to both HIV-1 viruses (Figure S8).

The ability of the wild-type and chimeric PGT antibodies to neutralize HIV-1 was assessed against the clade B Env-pseudoviruses SF162 and TRO.11 (Figure 4, Figure S5). Strong neutralization had been reported [13] against these two isolates for all PGT121-137 antibodies, except for PGT137 and PGT131 (on TRO.11), where the neutralization was relatively weak [13]. Observed neutralization correlated well with gp120 recognition for antibodies PGT121-131 from donors 17 and 36 (R^2^ = 0.739–0.943, *p*-value <0.001) (Figure S6), while those from antibodies PGT135-137 from donor 39 neutralized less well than expected based on gp120 recognition. Notably, chimeras containing the PGT123 light chain gave an intermediate level of neutralization with SF162 consistent with the gp120 recognition observed for these chimeras. In general, however, antibodies comprising intra-donor heavy/light chains gave medium to strong neutralization, whereas antibodies comprising inter-donor heavy/light chains showed weak neutralization.

### Heavy and Light Interface

Although expression levels of the chimeric antibodies were overall consistent, with an average of 58±10 µg/ml, suggesting no major disruption at the heavy/light chain interface, it is possible that for the antibodies to function, the interface between the heavy and the light chain should be clear of any potential clash. To investigate this possibility, we evaluated the amino acid changes at the heavy/light chain interface for structural compatibility (Table S1) [37]. The sequence analysis revealed that all changes were allowed in the Vgene region of the heavy chain but that there could be potential clash at position 34, 91 and 96 of the light chain that could result in nonfunctional chimeric antibodies (PGT121L has GLN, TRP and TRP in place of SER34, LEU91 and VAL96, respectively, for PGT128L) (Table S1). However superposition of variable regions of PGT121 and PGT128 does not show any particular clashes. Because it is difficult to predict germline for the CDR H3 region, we used modeling to evaluate this region, even though there were uncertainties in this loop region. The modeling indicated that changes in residue 100i in the CDR H3 could result in potential clash (although none were observed when variable regions of PGT121 and PGT128 were superimposed).

### Homology Modeling

**Figure 5:**
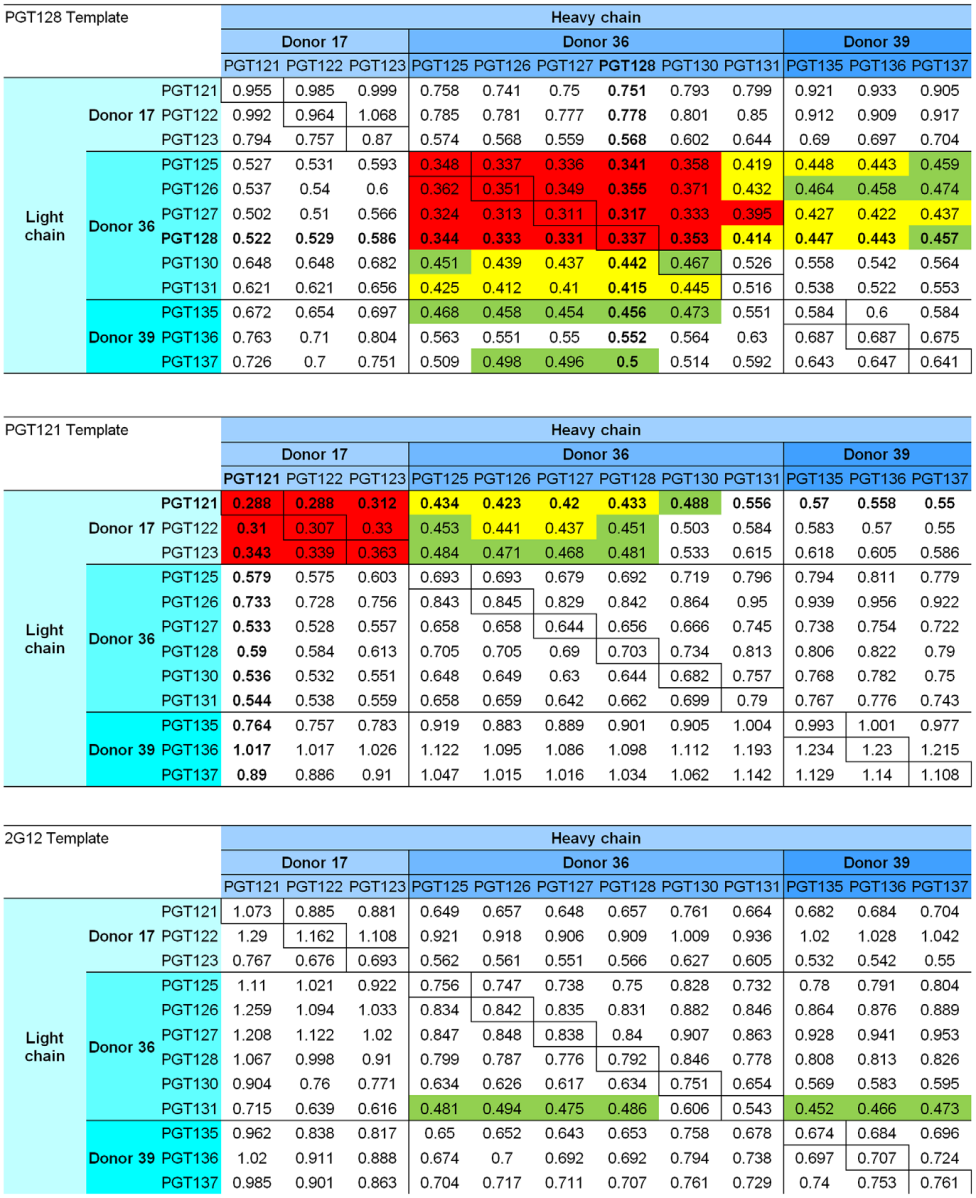
DFIRE scores of homology modeling of PGT121-137, based on PGT128, PGT121 and 2G12 template structures. Sequences alignment and homology modeling were carried out with sequences for PGT121-137 heavy and light chain and PGT128, PGT121 and 2G12 structural templates. Low scores indicate good homology modeling and were lowest for the PGT128 sequence modeled on the PGT128 structure (or PGT121 sequence modeled on PGT121 structure). DFIRE score <0.4 are shown in red, 0.4–0.45 in yellow and 0.45–0.5 in green.

We used structural modeling based on published PGT121-137 antibodies to predict structures of the different Mabs and evaluated the model quality with DFIRE scores [36]. We used known templates, PGT128 and 2G12 as well as the recently published PGT121, and obtained DFIRE scores (Figure 5). The lowest DFIRE scores were obtained for antibodies where the structural template was from the same donor as the threaded sequence (intra-donor homology modeling). This suggests that when DFIRE scores are low, the mode of recognition is likely the same. The homology modeling, however, did not provide as clear discrimination of mode of recognition as our heavy/light functional complementation. For example, with the PGT130-131 subset of PGT125-131 antibodies, the homology modeling indicates the PGT130-131 were as different in mode of recognition from PGT128 as the PGT135-137 (Figure 5); by contrast the functional complementation showed the light chains for PGT130-131 were able to complement the heavy chains of PGT125-128, and moreover that this was different from PGT135-137 (Figure 3). These results indicate that homology modeling can provide information on the mode of recognition, when the template antibody is known; however, the heavy/light functional complementation allows comparison of more diverse antibodies and also of antibodies that do not have a template structure.

## Discussion

The identification of dozens of broadly neutralizing antibodies in the last two years and the determination of the sequences for hundreds more presents an unprecedented wealth of information on how the human immune system can effectively neutralize HIV-1. Although each broadly neutralizing antibody represents a potential vaccine template, the very large number of potential templates represented by the newly identified antibodies outstrips the ability of vaccine designers to create immunogens that might elicit similar antibodies. One potential way to triage this design bottleneck would be to focus on antibodies with common modes of recognition that are elicited in multiple donors. In this way, vaccine designers can focus on recognition modalities that are more commonly elicited, and thus may be more easily “re-elicited”.

Implementation of such a “triage” step depends on the ability to determine the recognition mode for a particular antibody. An initial determination of recognition mode involves identification of the recognized HIV-1 envelope epitope. To a first approximation, only four sites on HIV-1 Env are recognized by the broadly neutralizing antibodies (the membrane-proximal region of gp41, the site of CD4-binding on HIV-1 gp120, and two glycosylated regions on HIV-1 gp120, characterized by N160 and N332, respectively), with each of the gp120 “sites-of-vulnerability” recognized by many of the newly identified broadly neutralizing antibodies. Sequence alignments, mutational analysis, and structural modeling can also provide clues to the mode of recognition, although the structural similarity between all antibodies, and the high divergence between antibodies with similar modes of recognition is well known; the VRC01-class of antibodies, for example, have the same mode of recognition, but may diverge in amino acid sequence by over 50% [11], [12]. More complete determination of the mode of antibody recognition can be accomplished by structural determination of the antibody in complex with its recognized epitope. All of the gp120-recognized epitopes for the broadly neutralizing antibodies thus far identified, however, are complex, requiring crystallization of at least a domain of gp120 with the antibody, and the crystallization of complexes of glycosylated gp120 with antibody has not kept pace with the rapid identification of new antibodies.

**Figure 6:**
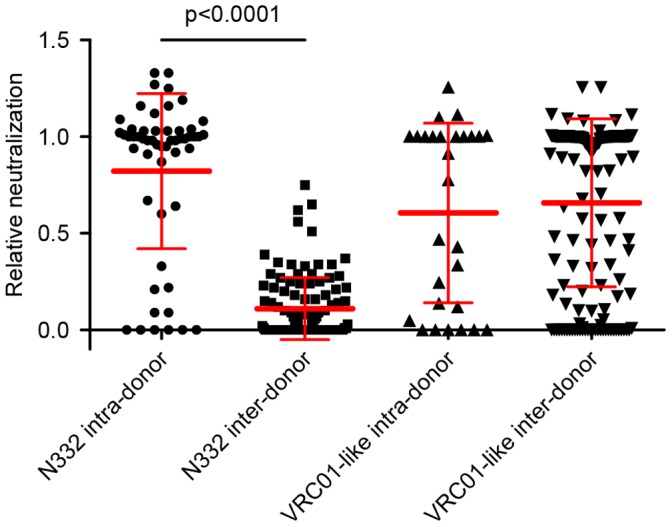
Relative interdonor complementation for N332-directed antibodies versus VRC01-like antibodies. Relative neutralization of inter and intra-donor chimeric antibodies. The neutralization of the chimeric antibodies relative to the average neutralization of the wild-type antibodies for the heavy and light chain components is plotted for the N332-directed antibodies (produced by 96-well microplate-formatted expression method) and VRC01-like antibodies (produced by small scale protein production method). The mean N332 intra-donor relative neutralization is 0.82±0.4 while the inter-donor mean is far lower at 0.11±0.16 revealing a functional complementation in the intra-donor chimeras that is not seen with the inter-donor antibodies (p<0.0001). Conversely the VRC01-like antibodies display similar results for intra (0.61±0.45) and inter-donor (0.66±0.43) complementation (p = 0.58). Neutralization values of VRC01-like chimeras were taken from 50 µg/ml concentration points used in previously published neutralization titers (31). Results for strains in which the wild-type neutralization was below 50% were excluded from the analysis.

An alternative means for assessing similarity in mode of recognition between two different antibodies involves the complementation of antibody function after swapping of heavy and light chains, and we previously showed such swapping of heavy and light chains between antibodies that have the same mode of recognition at the CD4-binding site to produce functional antibodies [11]. While the intradonor complementation was not quite to the level of the wild-type antibodies, substantial complementation was observed (Figure 6). Once the mode of antibody recognition has been determined for a set of reference antibodies, the mode of recognition for a new antibody relative to those of the reference set can then be determined by assessing the ability of heavy and light chains of the new antibody to complement functionally any of the reference set. This means of assessing similarity uses to advantage the determined sequences for many of the newly identified antibodies as well as the sensitive detection capabilities of ELISA and neutralization.

Structural modeling was also performed and does provide information about mode of recognition (when template antibody structures were available). This information, however, was substantially less robust than that obtained by functional complementation, and was only able to provide information on mode of recognition for closely related antibodies.

To express a matrix of heavy and light chains, we developed a 96-well transient transfection format. In general, 96-well microplate-formatted cell culture is widely used for various high-throughput cell-based assays, but is not often used for high-throughput protein expression. To facilitate high-throughput antibody function assays, we improved protein yield through optimization of cell growth, expression, and transient transfection protocols. The yield of antibodies prepared by this approach is as good as or better than those made by suspension cell culture-based protein production routinely used in our laboratory (Figure S2). This enabled the direct testing of the IgG supernatants in binding and neutralization assays.

We assessed the expressed antibodies for their recognition of gp120 and neutralization of HIV-1. For each HIV-1 strain, the intradonor-complemented heavy and light chains bound or neutralized well, whereas the interdonor-complemented antibodies did not. The level of inter-donor complementation for the N332-antibodies was substantially lower than that observed with the VRC01-like antibodies (Figure 6), despite similar degree of inter-donor antibody diversity (Figure S7). Although no effect on chimeric antibodies expression was observed, it is possible that mutations at the heavy/light chain interface could affect the complementation especially at position 34, 91 and 96 of the light chain (Table S1) [37]–[40].

Our functional complementation results indicate that each of the donors for the PGT121-137 antibodies recognizes the N332-region of HIV-1 gp120 in a different way. This is somewhat unexpected as many of the PGT121-137 antibodies recognize glycan in similar ways [13], and the heavy chains for antibodies PGT125-137 from two of the donors have exactly the same predicted V- and J-gene origins [13]. This observation is, nonetheless, confirmed by the newly described PGT121 structure [26]; although this new PGT121 structure was not determined in complex with HIV-1 gp120, the glycan recognition was clearly different from that of PGT128.

The diversity of recognition for the N332-directed antibodies contrasts with that of the CD4-binding site, where a number of antibodies converge on the same “VRC01-like” mode of heavy chain mimicry of CD4. It is not clear if the sequence variation in PGT121-137 could be an indication of the different modes of recognition since this is not the case for the VRC01-like antibodies. The diversity of conformations adopted by the N332 epitope may be larger than the highly restricted epitope at the CD4-binding site [41], which might lead to the diversity in binding modes suggested here. Whether the diversity of N332-directed modes of recognition represents a boon or a cautionary note to vaccine designers remains to be determined. Characterization of the mode of recognition, nonetheless, represents a critical step in the antibody-template model of vaccine development – a step perhaps eased by the efficient means of functional complementation described here.

## Supporting Information

Figure S1 **Expression of a complete matrix of PGT121-137 heavy and light chains (µg/mL).** The titer data were generated as compared and normalized to the control titer (1.1 µg/mL) from three non-DNA transfected wells (supernatants without an antibody IgG). The average titer of entire expressed PGT antibodies is 58.0 µg/ml with 9.96 SD. The supernatant with expressed VRC01 was used as positive control (titer 171.6 µg/mL).
(TIF)

Figure S2 **High antibody expression levels achieved through optimization of cell growth, expression, and transfection protocols.** 96-well microplate-formatted transient gene transfection technology was used for high-throughput antibody expression. The titer of VRC01 IgG production (11.1 µg/ml) (a), was increased by 8.7-fold (96.3 µg/ml) through optimization of cell growth and nutrient feed/transgene expression enhancers (b), and was further increased 1.7-fold (163.9 µg/ml) after optimization of transient transfection parameters (c).The titers of VRC01 antibody produced by the approach in (c) are even higher than those made by suspension cell culture-based protein production (140.8 µg/ml) in (d) routinely used in our laboratory, and allow high throughput neutralization assays and other applications. In (a), (b) and (c), each average titer was derived from three individual wells, and in (d) the average yield was from three VRC01 production lots. Control levels of expression and biological properties are shown in Figure S8.
(TIF)

Figure S3 **Similar expression levels and quality of light chains and heavy chains pairing in inter-donor and intra-donor chimeric Abs.** The pairs of light chains and heavy chains between inter-donors PGT121-H/PGT128-L, PGT121-H/PGT135-L, PGT128-H/PGT121-L, PGT135-H/PGT121-L, intra-donors PGT121-H/PGT122-L, PGT122-H/PGT121-L and control donor PGT121-H/PGT121-L were expressed at small scales (250 ml/expression) in HEK 293F cells with the same condition used in 96-well microplate-formatted transient transgene expression. The expression levels of Abs were estimated by measurement of IgG with NanoDrop 2000c spectrophotometer (Thermo Scientific, Wilmington, DE), and quality of Abs analyzed with SDS-PAGE gels. The similar expression levels of Abs existed among inter-donors or intra-donors. No “unassembled” forms of expressed antibodies were detected.
(TIF)

Figure S4 **ELISA binding to HIV-1 gp120 by PGT121-137 antibodies and chimeric variants in units of OD_450_ nm.** Strong binding is coded in red (>1.0 OD450 nm), intermediate in yellow (0.5–1.0 OD450 nm) and weak in green (0.3–0.5 OD450 nm). Each reported value is the average from three individual measurements.
(TIF)

Figure S5 **Neutralization by PGT121-137 antibodies and chimeric variants.** Strong neutralization is coded in red (>90%), intermediate in yellow (75–90%) and weak in green (50–75%). Neutralization data were generated in single neutralization experiments, with each test sample analyzed in duplicate to determine the percent decrease in virus growth (neutralization) as compared and normalized to the control wells without antibody expression (defined as 0% neutralization). Each antibody was assayed at a 1∶5 final dilution with virus added.
(TIF)

Figure S6 **Correlation of BaL gp120 recognition relative to HIV-1 neutralization.** Correlation of BaL gp120 recognition (y axis) versus neutralization (x axis) of HIV-1 SF162.LS (A) and HIV-1 TRO.11 (B) by donor. The data of the BaL gp120 recognition and HIV-1 neutralization are plotted and analyzed with GraphPad Prism 5. The linear regression, correlation coefficient (R^2^), and P value are presented in the graph. The significance of correlation was observed in donors 17 (R^2^ = 0.875 for HIV-1 SF162.LS, and 0.943 for TRO.11; P<0.001), donor 36 (R^2^ = 0.892 for HIV-1 SF162.LS, and 0.739 for TRO.11; P<0.001), and donor 39 within the neutralization of HIV-1 TRO.11 (R^2^ = 0.345; P<0.001). PGT135-137 antibodies do not neutralize HIV-1 SF162.LS.
(TIF)

Figure S7 **Superposed heavy and light chain nucleotide sequence diversity for four CD4-binding site-directed antibodies and PGT121-137 antibodies.** Neighbor-joining (NJ) phylogenetic method is used to calculate the dendrograms for both sets of antibodies, with CD4-binding site-directed antibody dendrogram shown in red and PGT121-137 antibody dendrogram shown in light gray. Donor and germline gene (V and J) information are also labeled for CD4-binding site-directed antibodies.
(TIF)

Figure S8 **Expression levels, binding, and neutralization for 17b, VRC01 & VRC-CH31.** Strong binding is coded in red (>1.0 OD_450_ nm), intermediate in yellow (0.5–1.0 OD_450_ nm) and weak in green (0.3–0.5 OD_450_ nm). Strong neutralization is coded in red (>90%), intermediate in yellow (75–90%) and weak in green (50–75%).
(TIF)

Table S1 **Heavy and light chain interface residue and structural effect in complementation.**
(PDF)
