# Supplementary figures and images for: N332-Directed Broadly Neutralizing Antibodies Use Diverse Modes of HIV-1 Recognition: Inferences from Heavy-Light Chain Complementation of Function
Source: PLoS One. 2013 Feb 19;8(2):e55701. doi: 10.1371/journal.pone.0055701 (PMC3576407; doi:10.1371/journal.pone.0055701)

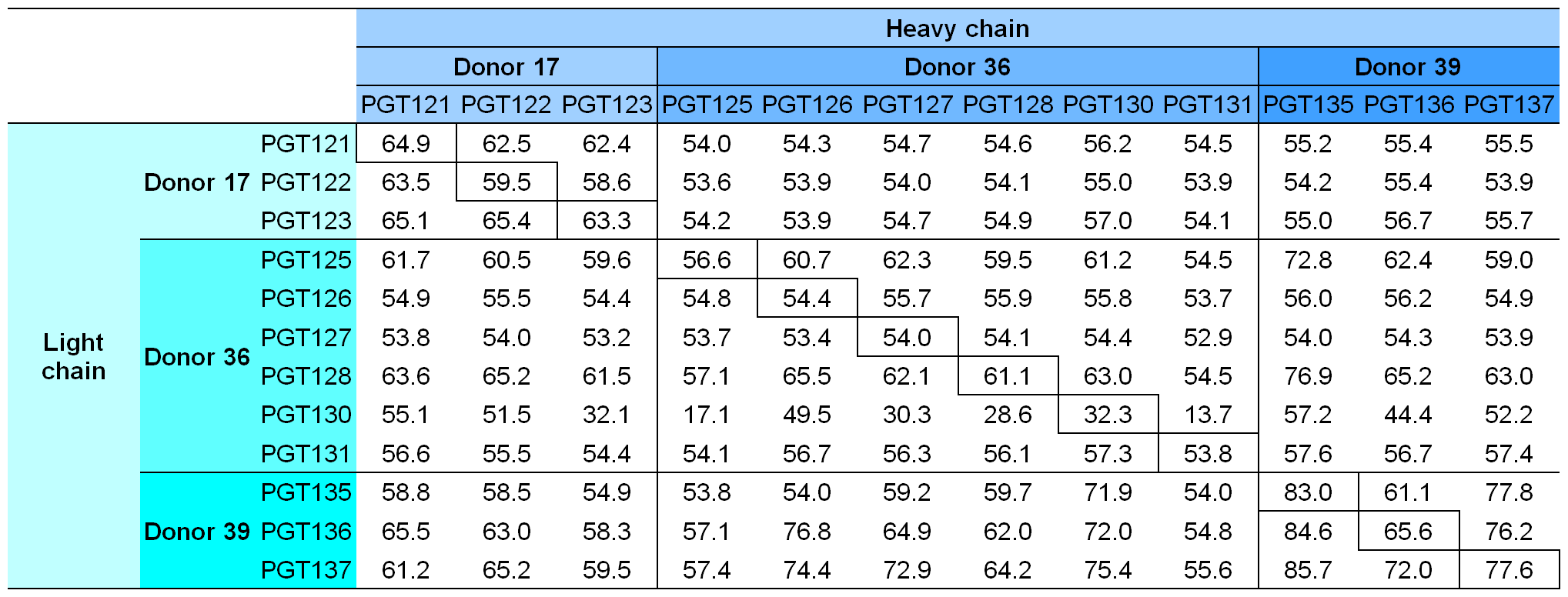

Supplement: Figure S1 — Expression of a complete matrix of PGT121-137 heavy and light chains (µg/mL). The titer data were generated as compared and normalized to the control titer (1.1 µg/mL) from three non-DNA transfected wells (supernatants without an antibody IgG). The average titer of entire expressed PGT antibodies is 58.0 µg/ml with 9.96 SD. The supernatant with expressed VRC01 was used as positive control (titer 171.6 µg/mL). (TIF) [file pone.0055701.s001.tif]

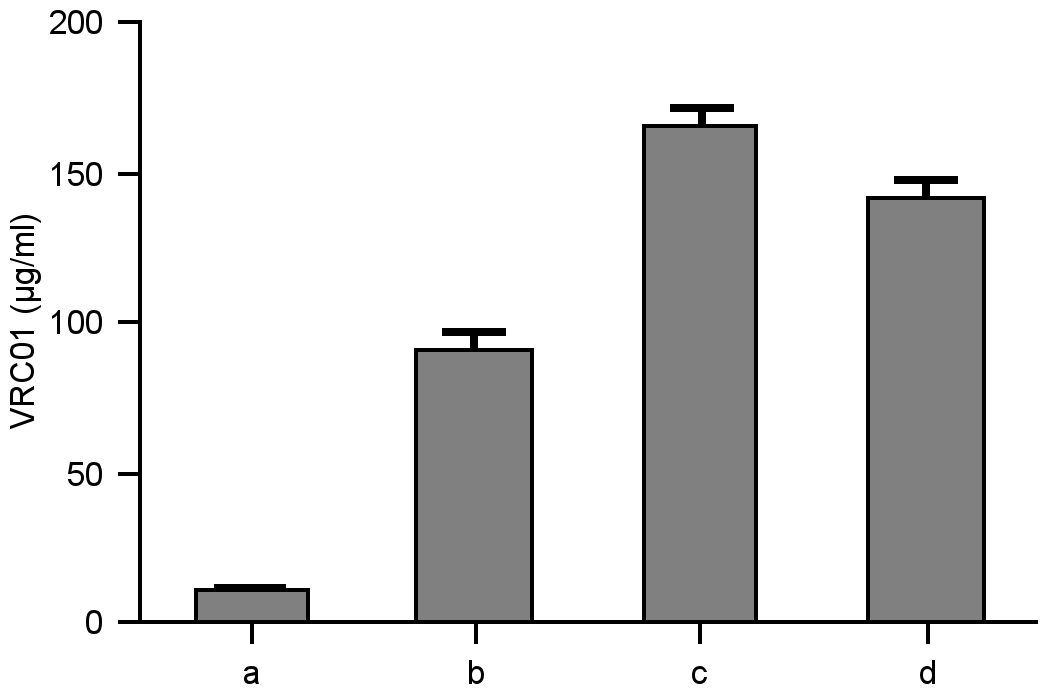

Supplement: Figure S2 — High antibody expression levels achieved through optimization of cell growth, expression, and transfection protocols. 96-well microplate-formatted transient gene transfection technology was used for high-throughput antibody expression. The titer of VRC01 IgG production (11.1 µg/ml) (a), was increased by 8.7-fold (96.3 µg/ml) through optimization of cell growth and nutrient feed/transgene expression enhancers (b), and was further increased 1.7-fold (163.9 µg/ml) after optimization of transient transfection parameters (c).The titers of VRC01 antibody produced by the approach in (c) are even higher than those made by suspension cell culture-based protein production (140.8 µg/ml) in (d) routinely used in our laboratory, and allow high throughput neutralization assays and other applications. In (a), (b) and (c), each average titer was derived from three individual wells, and in (d) the average yield was from three VRC01 production lots. Control levels of expression and biological properties are shown in Figure S8. (TIF) [file pone.0055701.s002.tif]

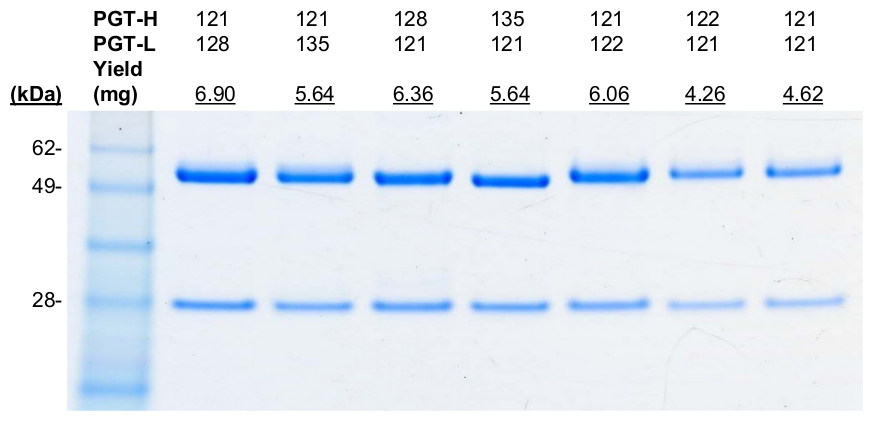

Supplement: Figure S3 — Similar expression levels and quality of light chains and heavy chains pairing in inter-donor and intra-donor chimeric Abs. The pairs of light chains and heavy chains between inter-donors PGT121-H/PGT128-L, PGT121-H/PGT135-L, PGT128-H/PGT121-L, PGT135-H/PGT121-L, intra-donors PGT121-H/PGT122-L, PGT122-H/PGT121-L and control donor PGT121-H/PGT121-L were expressed at small scales (250 ml/expression) in HEK 293F cells with the same condition used in 96-well microplate-formatted transient transgene expression. The expression levels of Abs were estimated by measurement of IgG with NanoDrop 2000c spectrophotometer (Thermo Scientific, Wilmington, DE), and quality of Abs analyzed with SDS-PAGE gels. The similar expression levels of Abs existed among inter-donors or intra-donors. No “unassembled” forms of expressed antibodies were detected. (TIF) [file pone.0055701.s003.tif]

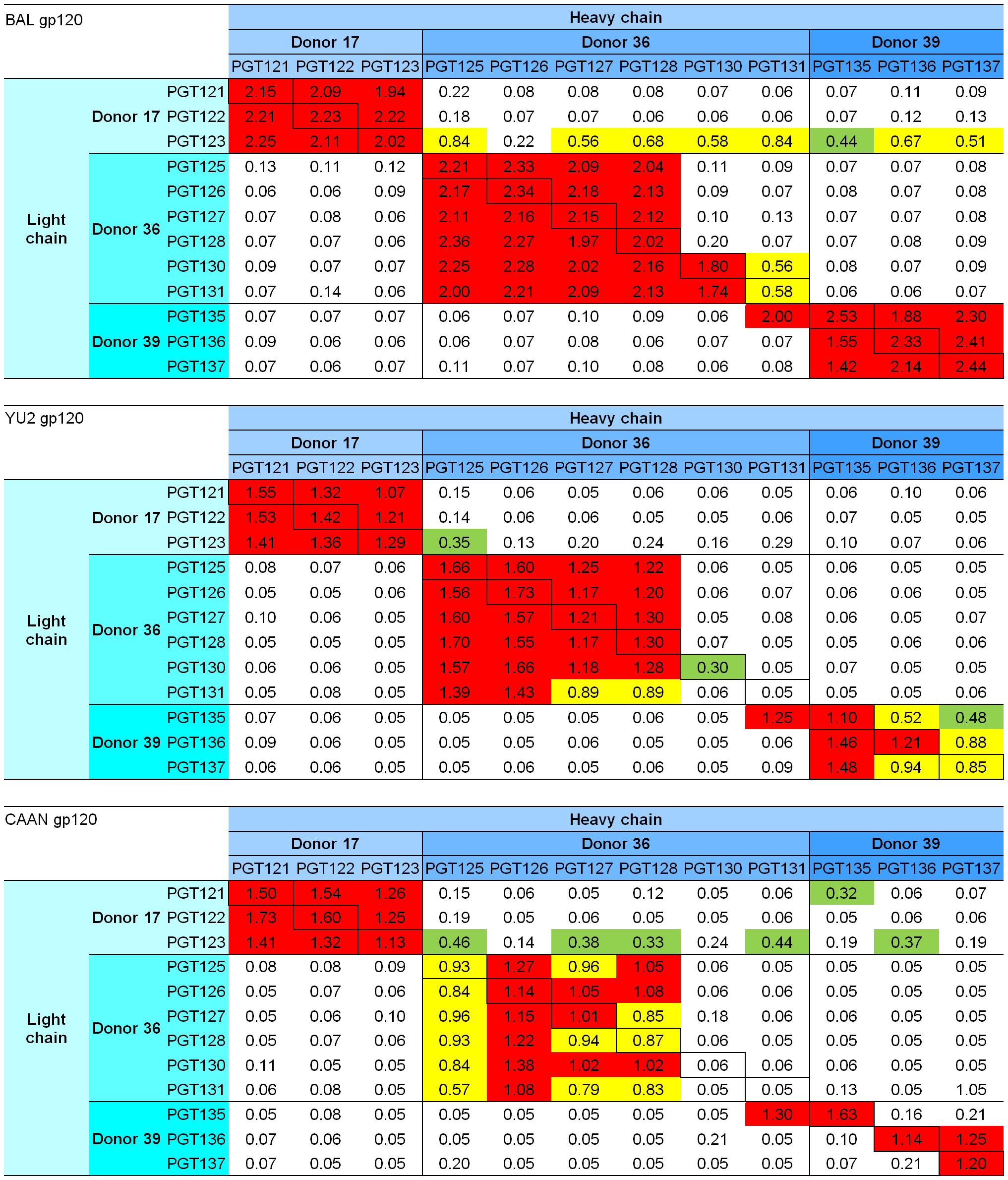

Supplement: Figure S4 — ELISA binding to HIV-1 gp120 by PGT121-137 antibodies and chimeric variants in units of OD450 nm. Strong binding is coded in red (>1.0 OD450 nm), intermediate in yellow (0.5–1.0 OD450 nm) and weak in green (0.3–0.5 OD450 nm). Each reported value is the average from three individual measurements. (TIF) [file pone.0055701.s004.tif]

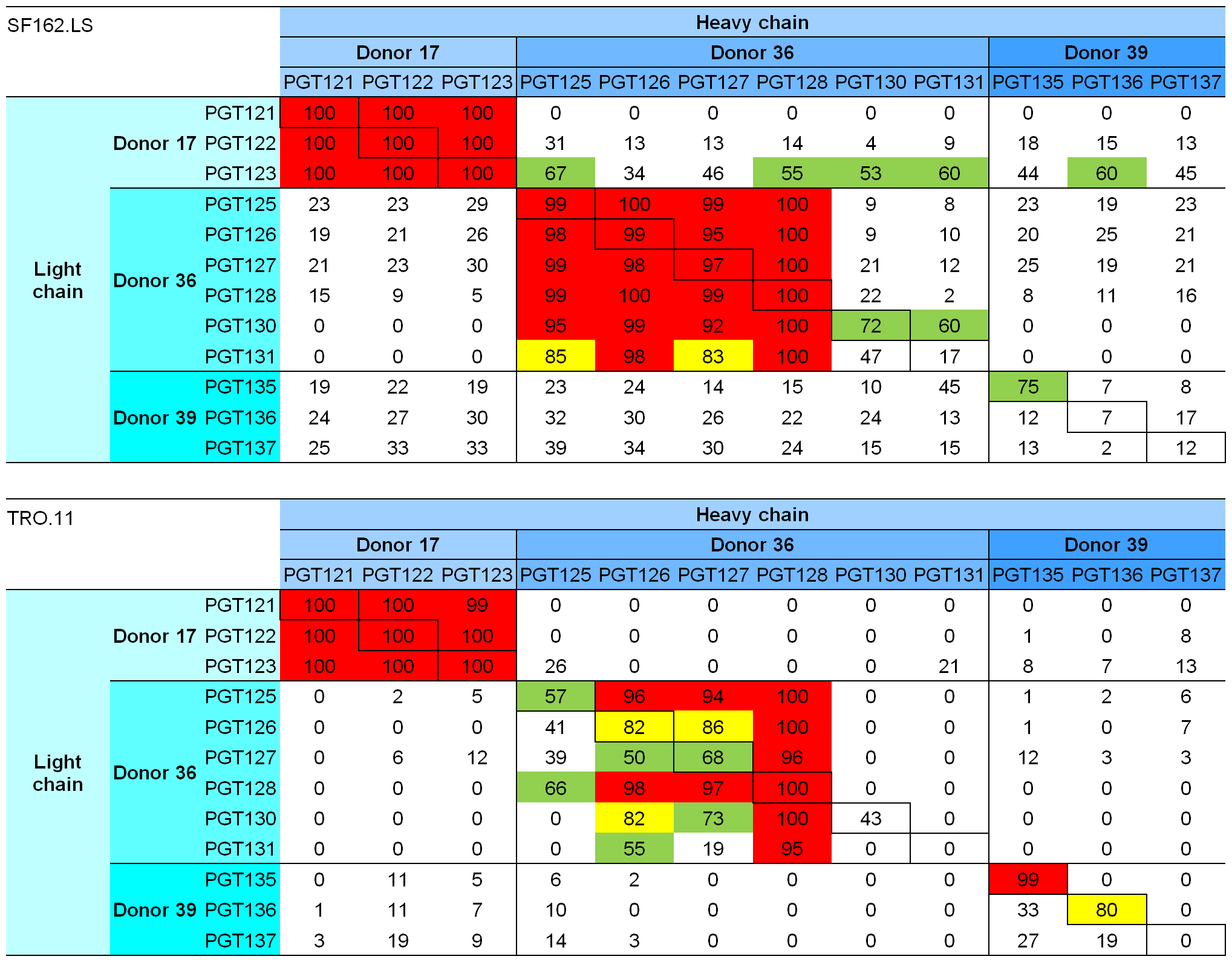

Supplement: Figure S5 — Neutralization by PGT121-137 antibodies and chimeric variants. Strong neutralization is coded in red (>90%), intermediate in yellow (75–90%) and weak in green (50–75%). Neutralization data were generated in single neutralization experiments, with each test sample analyzed in duplicate to determine the percent decrease in virus growth (neutralization) as compared and normalized to the control wells without antibody expression (defined as 0% neutralization). Each antibody was assayed at a 1∶5 final dilution with virus added. (TIF) [file pone.0055701.s005.tif]

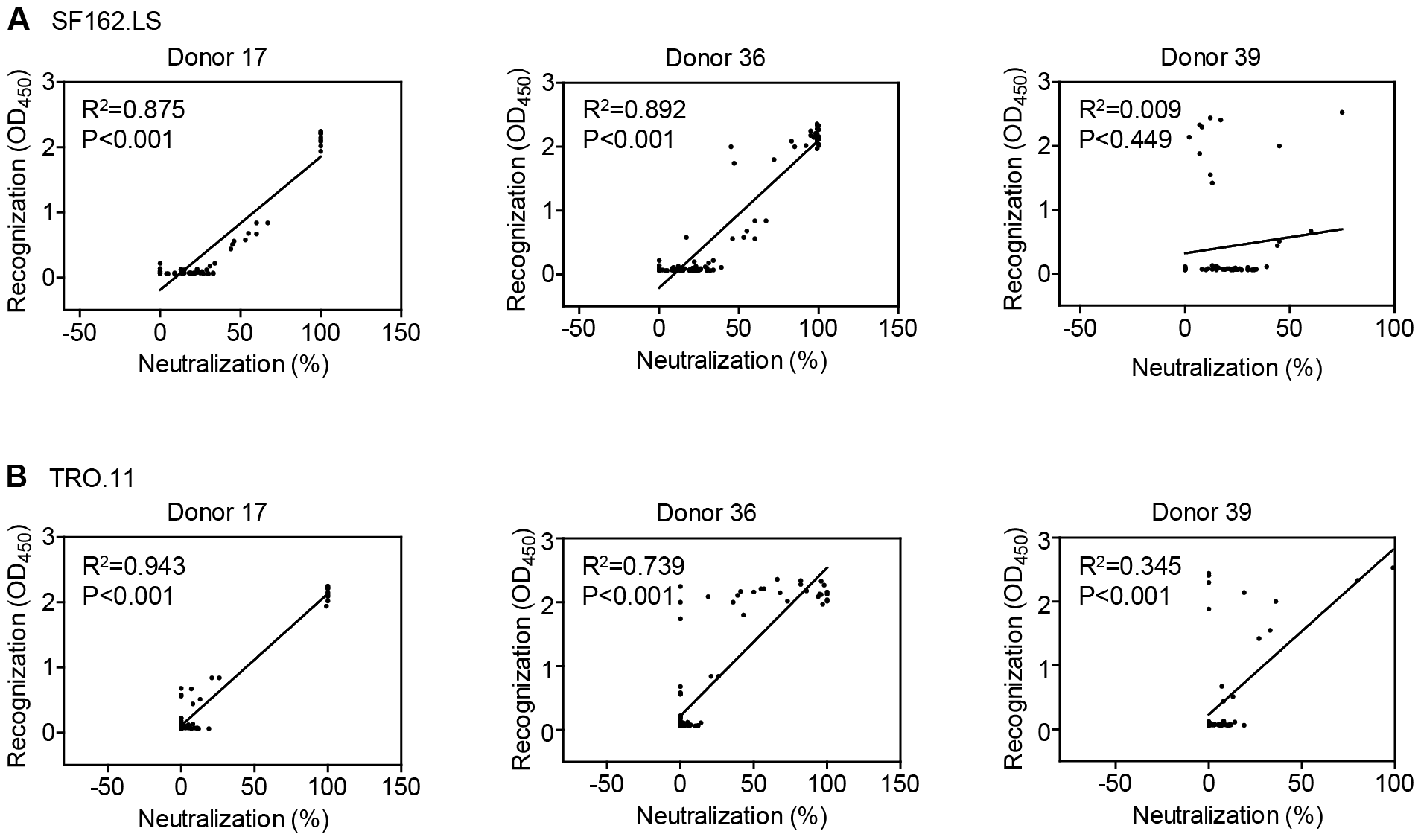

Supplement: Figure S6 — Correlation of BaL gp120 recognition relative to HIV-1 neutralization. Correlation of BaL gp120 recognition (y axis) versus neutralization (x axis) of HIV-1 SF162.LS (A) and HIV-1 TRO.11 (B) by donor. The data of the BaL gp120 recognition and HIV-1 neutralization are plotted and analyzed with GraphPad Prism 5. The linear regression, correlation coefficient (R2), and P value are presented in the graph. The significance of correlation was observed in donors 17 (R2 = 0.875 for HIV-1 SF162.LS, and 0.943 for TRO.11; P<0.001), donor 36 (R2 = 0.892 for HIV-1 SF162.LS, and 0.739 for TRO.11; P<0.001), and donor 39 within the neutralization of HIV-1 TRO.11 (R2 = 0.345; P<0.001). PGT135-137 antibodies do not neutralize HIV-1 SF162.LS. (TIF) [file pone.0055701.s006.tif]

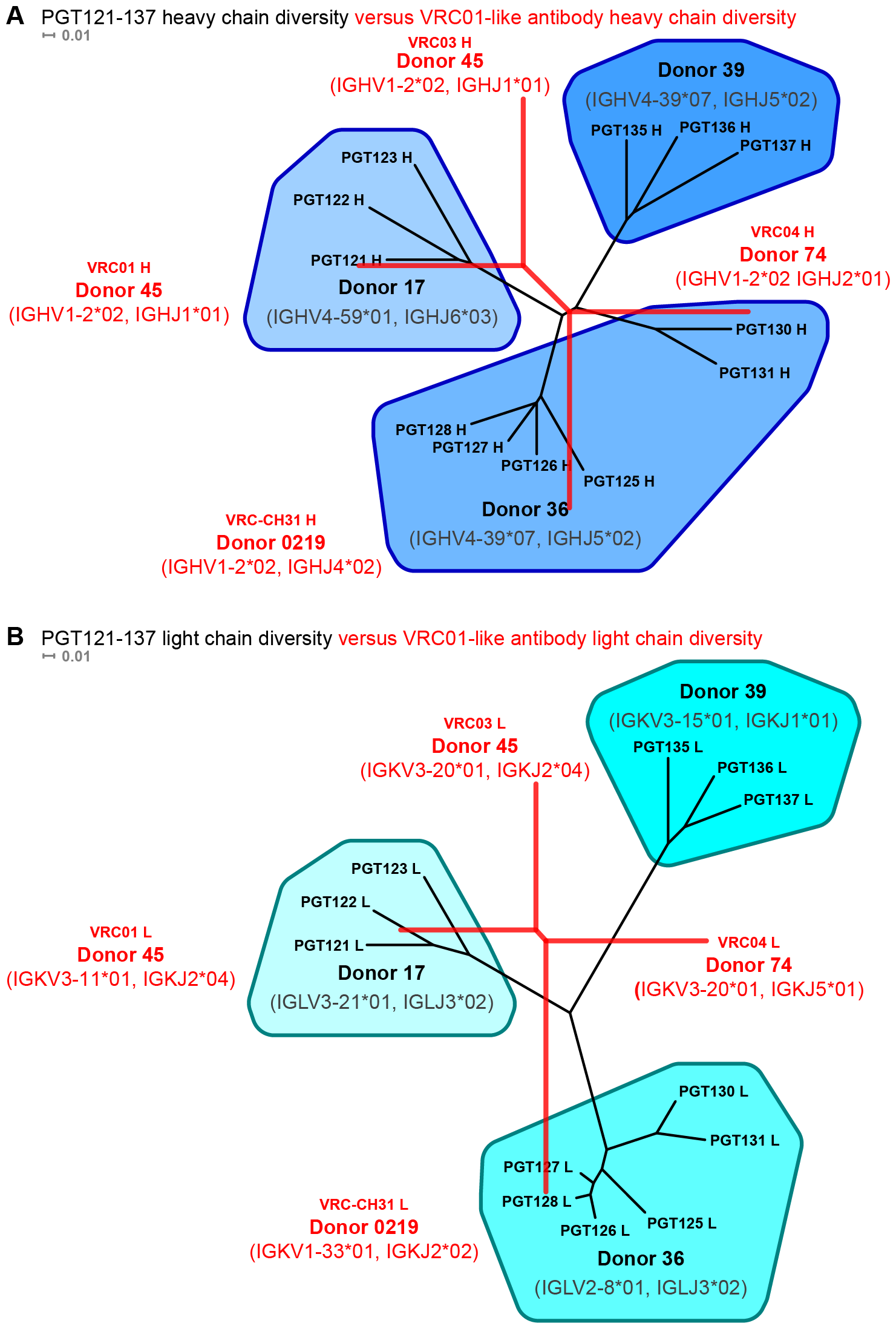

Supplement: Figure S7 — Superposed heavy and light chain nucleotide sequence diversity for four CD4-binding site-directed antibodies and PGT121-137 antibodies. Neighbor-joining (NJ) phylogenetic method is used to calculate the dendrograms for both sets of antibodies, with CD4-binding site-directed antibody dendrogram shown in red and PGT121-137 antibody dendrogram shown in light gray. Donor and germline gene (V and J) information are also labeled for CD4-binding site-directed antibodies. (TIF) [file pone.0055701.s007.tif]

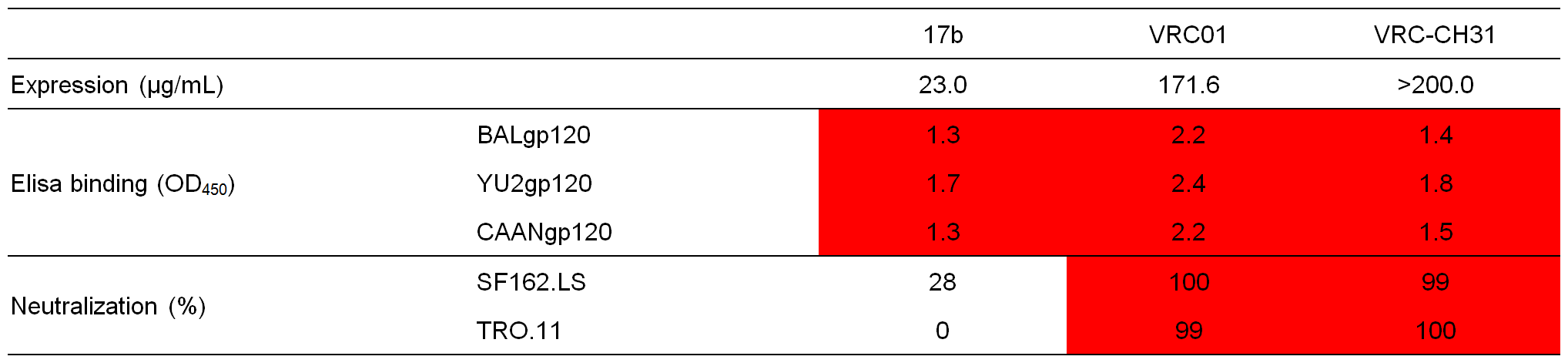

Supplement: Figure S8 — Expression levels, binding, and neutralization for 17b, VRC01 & VRC-CH31. Strong binding is coded in red (>1.0 OD450 nm), intermediate in yellow (0.5–1.0 OD450 nm) and weak in green (0.3–0.5 OD450 nm). Strong neutralization is coded in red (>90%), intermediate in yellow (75–90%) and weak in green (50–75%). (TIF) [file pone.0055701.s008.tif]
