# Supplementary material for: N332-Directed Broadly Neutralizing Antibodies Use Diverse Modes of HIV-1 Recognition: Inferences from Heavy-Light Chain Complementation of Function
Source: PLoS One. 2013 Feb 19;8(2):e55701. doi: 10.1371/journal.pone.0055701 (PMC3576407; doi:10.1371/journal.pone.0055701)
Supplement: Table S1 — Heavy and light chain interface residue and structural effect in complementation. (PDF) [file pone.0055701.s009.pdf]

Table S1. Heavy and light chain interface residue and structural effect in complementation.

Residue	PGT121-123	PGT125-131	PGT135-137	Conserved substitution	Structural effect in complementation
H35	**S**	G	G	Yes	None
H37	I	V	V/I	Yes	None
H39	R/Q	Q	H/Q	Yes	None
H45	L/P	L/P	L	No	None, functional complementation
H47	W	W	W	Yes	None
H91	Y/F	Y/H/F	F/Y	Yes	None
H93	A	A/V	A/V	Yes	None
H95	T/A	F/S	H	No	Possible incompatibility, not a major interface residue
H101	D	D/S/Y	D	No	None, functional complementation
H103	W	W	W	Yes	None
L32	S/A	F	N	Yes	Possible incompatibility, not a major interface residue
L34	Q/I	S	A	Yes	Could cause major clashes
L36	Y	Y	Y	Yes	None
L38	H/Q	Q	Y/L/K	No	None, functional complementation
L43	A/P	A	S	Yes	None
L44	P	P	P	Yes	None
L46	L	L	L	Yes	None
L50	N	G/D/E	E/D	No	Possible incompatibility, not a major interface residue
L86	Y	Y	Y	Yes	None
L87	Y	F	Y/F	Yes	None
L89	H	S/G	Q	Yes	Possible incompatibility, not a major interface residue
L91	W/Y	L	Y	No	Could cause major clashes
L96	W	V/I	R	No	Could cause major clashes
L98	F	F	F	Yes	None
